# Dynamic trajectories of life-threatening mass effect in patients with large middle cerebral artery stroke

**DOI:** 10.21203/rs.3.rs-3594179/v1

**Published:** 2023-11-20

**Authors:** Charlene Ong, Qiuxi Huang, Ivy Kim, Jack Pohlmann, Stefanos Chatzidakis, Benjamin Brush, Yihan Zhang, Yili Du, Leigh Ann Mallinger, Emelia J. Benjamin, Josée Dupuis, David Greer, Stelios Smirnakis, Ludovic Trinquart

**Affiliations:** Boston University Medical Campus; Boston University School of Public Health; Boston Medical Center; Boston Medical Center; Brigham and Women’s Hospital; New York University Medical Center: NYU Langone Health; Boston Medical Center; Boston University School of Medicine: Boston University Chobanian & Avedisian School of Medicine; Boston University School of Medicine: Boston University Chobanian & Avedisian School of Medicine; Boston University School of Medicine: Boston University Chobanian & Avedisian School of Medicine; Boston University School of Public Health; Boston University School of Medicine: Boston University Chobanian & Avedisian School of Medicine; Brigham and Women’s Hospital; Tufts Medical Center

## Abstract

**Background:**

Life-threatening, space-occupying mass effect due to cerebral edema and/or hemorrhagic transformation is an early complication of patients with middle cerebral artery (**MCA**) stroke. Little is known about longitudinal trajectories of laboratory and vital signs leading up to radiographic and clinical deterioration related to this mass effect.

**Methods:**

We curated a granular retrospective dataset of 635 patients with large middle cerebral artery (**MCA**) stroke totaling 108,547 data points for repeated measurements of 10 covariates, and 40 time-independent covariates. We assessed longitudinal trajectories of the 10 longitudinal variables during the 72 hours preceding three outcomes representative of life-threatening mass effect: midline shift (**MLS**) ≥5mm, pineal gland shift (**PGS**) >4mm, and decompressive hemicraniectomy (**DHC**). We used a “backward looking” trajectory approach. Patients were aligned according to the time of outcome occurrence and the trajectory of each variable was assessed prior to that outcome by accounting for both cases and non-cases.

**Results:**

Of 635 patients, 49% were female, and mean age was 69 years. Thirty five percent of patients had MLS ≥ 5mm, 24.1% had PGS >4mm, and DHC occurred in 10.7%. For the three outcomes of interest, backward-looking trajectories showed mild increases in white blood cell count (10 up to 11 K/UL within 72 hours), temperature (up to half a degree within 24 hours), and sodium (1–3 mEq/L within 24 hours) leading up to outcomes. We also observed a decrease in heart rate (75 – 65 beats per minute) 24 hours prior to DHC.

**Conclusions:**

Univariable longitudinal profiling showed that temperature, white blood cell count, and sodium increase prior to radiographic and clinical indicators of space-occupying mass effect. These findings will inform development of multivariable dynamic risk models to aid prediction of life-threatening space-occupying mass effect.

## Introduction

Life-threatening, space-occupying mass effect caused by cerebral edema with or without hemorrhagic transformation is a severe complication of large supratentorial ischemic stroke. It most often occurs within the first week after injury. Identifying patients at risk for developing clinically significant mass effect is essential to guide management. Increased stroke volume, decreased glucose levels, and early midline shift (**MLS**) or basal cistern effacement within 24 hours of admission are associated with increased risk of death or need for DHC due to space-occupying mass effect.^1–3^ However, baseline information is rarely sufficient to inform individual management decisions. Instead, close clinical monitoring to identify signs of deterioration, often confirmed by radiographic imaging, is used to trigger medical or surgical therapies.

Current standards in clinical modeling use static models at admission or within 24 hours to predict events that often occur by discharge or beyond.^1,4^ However, better methods are needed to tailor risk prediction and subsequent management decisions precisely to individual patients using dynamic information that changes over the first few days of injury. Assessing longitudinal trajectories of physical, laboratory, and radiographic assessments leading up to space-occupying mass effect would improve our understanding of changes in dynamic multimodal measurements that precede and may signal imminent neurological decline. Univariable longitudinal profiling also serves as a first step towards developing multivariable dynamic risk prediction models that can update predictions with new information and better reflect how clinicians currently monitor patients.

In this study, we created a curated longitudinal dataset of 635 patients with large middle cerebral artery (**MCA**) stroke totaling 108,547 data points for repeated measurements of 10 covariates, and 40 time-independent covariates. We focused on three outcomes indicative of space-occupying mass effect: MLS ≥ 5mm,^5–8^ pineal gland shift (**PGS**) > 4mm,^9^ and decompressive hemicraniectomy (**DHC**).^3^ We derived longitudinal trajectories for 10 variables leading up to each of these life-threatening, space occupying mass effect outcomes. We used a novel “backward looking” statistical approach^5^ in which we aligned trajectories by outcome to better identify clinically relevant patterns up to 72 hours before life-threatening space-occupying mass effect.

## Methods

We prepared this report according to Strengthening the Reporting of Observational Studies in Epidemiology (**STROBE**) reporting guidelines (Supplementary Methods).

### Data sources

**Figure 1:**
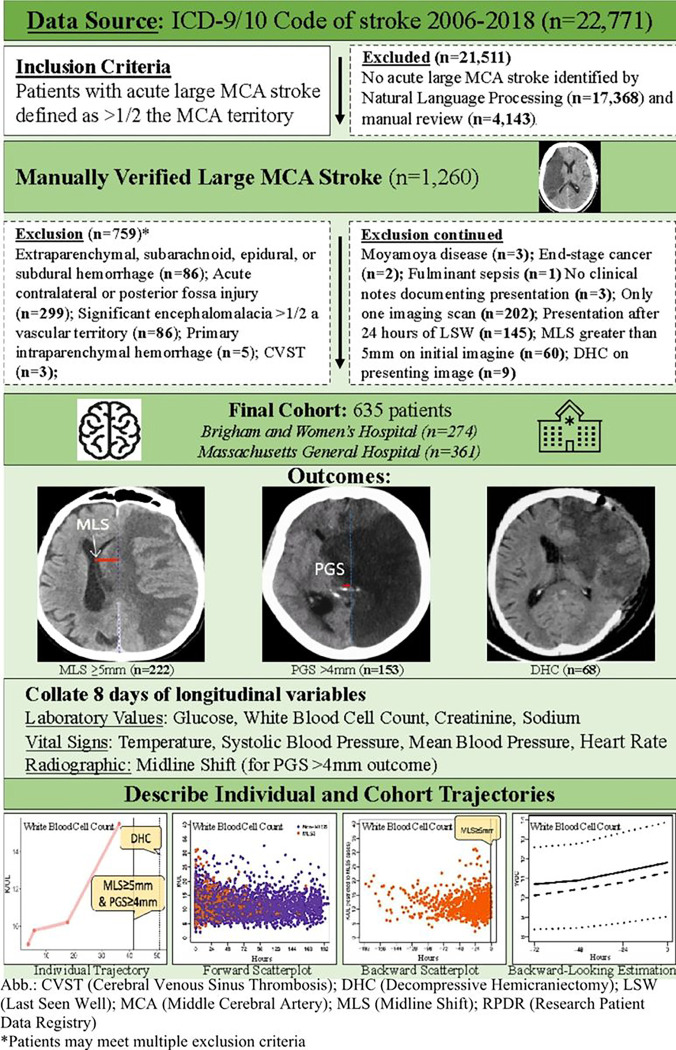
Inclusion/Exclusion Criteria and Study Schematic

We conducted a two-center retrospective cohort study of adult patients with acute ischemic MCA stroke from the Brigham and Women’s Hospital (**BWH**) and Massachusetts General Hospital (**MGH**) admitted between 2006 and 2021 who presented within 24 hours of last-seen-well. We first extracted patients using ICD-9 and ICD-10 codes for ischemic stroke between 2006–2021. We used a Natural Language Processing algorithm to identify patients with acute MCA stroke.^10^ We confirmed stroke size ≥ 1/2 of the MCA territory by trained team members using manual review of images. Full exclusion criteria and study schematic are detailed in Fig. 1. We used longitudinal data available within eight days of admission and censored for our outcome, and death or discharge from the hospital.

### Data Ascertainment and Processing

We collected demographic, laboratory, radiographic, procedural, clinical notes, and outcome data from the electronic medical record using the Research Data Patient Registry (**RPDR**) and Enterprise Data Warehouse.

To curate structured data from our local repositories, we first accrued structured and unstructured data from RPDR, and employed a series of decision rules. Medical history was obtained by using ICD-9 and ICD-10 diagnosis codes present on admission (except for stroke) or in any other prior encounter available from RPDR (Supplementary Methods). Last seen well was determined by manual review of clinical notes. Two team members (SK, CJO) reviewed all clinical notes and discussed/reached consensus for times in which LSW as unclear. NIHSS was extracted from the history and/or physical exam. For the cases where admission NIHSS was not explicitly stated in the patient’s admission record, it was inferred based on the available history and physical exam by a rater who was previously trained and certified in the administration of the NIHSS (LC).^5 11^ Acute stroke treatments including chemical thrombolysis and mechanical thrombectomy (**MT**), MT time, DHC occurrence and time were ascertained through review of all available clinical and/or procedural notes. Procedures and keywords used for reviewing notes for LSW, NIHSS and stroke treatments are available in the Supplementary Methods.

Laboratory values were obtained through structured RPDR database. The value closest to stroke time was defined as the value within eight hours before and up to 36 hours after the designated admission (or in the case of in-patient stroke, recognition) time. Vital signs were available from patients post-2015 (when EPIC became the electronic medical record). Admission vital signs for patients prior to 2015 were taken from chart review of the History and Physical. To remove clearly erroneous lab or vital sign errors due to incorrect chart entry, each longitudinal measurement was assigned a Z-score based on the distribution of the data in the full cohort. Any measurement with an absolute Z-score greater than or equal to three, or 25% change from a preceding value, was flagged as a potential erroneous measurement. The principal investigator (CJO) examined each patient’s flagged laboratory/vital sign trajectories and determined which outlying values fit in the context of their hospital course and 14 data points from eight patients were removed (< 0.01% of longitudinal measurements).

Radiology variables were reviewed by a trained MD (SC) and underwent secondary review by CJO or BB. For cases in which there was disagreement, discrepancies were adjudicated by an additional reviewer (BB, CJO) to reach consensus. Further details on inter-rater reliability is included in the Supplementary Methods. Radiology variables included The Alberta Stroke Program Early CT Score (**ASPECTS**), MLS, and PGS. A review of 10% of images resulted in 83.9% agreement for high, medium, and low ASPECTS (10 − 8, 7 − 4, 3 − 0). The average of the lateral and medial boundary was used to calculate MLS used in analysis. Mean absolute error of MLS and PGS between raters and radiographic reports was 0.19 and 0.36 mm respectively in 10% of the data. Percent agreement between trained team members (SC, CJO) on a 10% sample was 92.5% for hemorrhage.

Further information on data collection and data quality for our variables, outcomes of interest, and missing data are included in the Supplementary Methods. The study was approved by the Massachusetts General-Brigham Institutional Review Board.

### Outcomes

We focused on three outcomes indicative of life threatening, space occupying mass effect: the first occurrence of MLS ≥ 5mm, the first occurrence of PGS > 4mm, and DHC. MLS ≥ 5mm is indicative of severe swelling associated with decreased consciousness ^12^ and need for treatment,^13^ and has been used as a marker of malignant space-occupying mass effect (with or without other clinical events) in prior studies.^7^ Because edema can occur in rare instances even over a week after stroke,^9^ we chose to evaluate patients through 8 days, or 192 hours. Because the natural progression of MLS can extend beyond 5mm and progress to even more extreme anatomic shifts, we also modeled trajectories leading up to PGS > 4mm, as PGS > 4mm has been previously been associated with diminished consciousness,^12^ asymmetric pupil reactivity,^14^ and higher mortality.^9^ Finally, we used DHC as a clinical outcome as surgery is often triggered by decreased arousal and/or neurologic deterioration. If the first occurrence of MLS ≥5mm or PGS > 4mm happened after DHC, and was not coded as an outcome. To examine when outcomes occurred, we estimated the cumulative incidence function of MLS ≥ 5mm, PGS >4mm, and DHC separately, all accounting for the competing risk of death by using the Aalen-Johansen estimator.^15^ We reported the cumulative incidences with 95% confidence interval at clinically relevant follow-up times.

### Longitudinal Exposures and Covariates

The dynamic longitudinal variables we chose to investigate included common laboratory and vital signs, some of whose baseline values have prior associations with cerebral edema in the literature.^1,3,16,17^ They included glucose, sodium, creatinine, and white blood cell count (**WBC**). On a subset of patients with available data from 2015 onward (N = 166), we also reviewed longitudinal data on blood pressure (systolic, diastolic, mean arterial), heart rate, and temperature. For the outcome of PGS > 4mm, we also examined the trajectory of MLS. Median times between two consecutive measurements (restricted before outcomes) were 5.4 [25th, 75th percentile 2.3, 8.3], 8.9 [6.0, 19.6], 11.3 [6.6, 22.9] and 20.9 [9.3, 24.4] hours for glucose, sodium, creatinine, and WBC, respectively. Blood pressure and heart rate were collected at a median of every one hour, temperature every two hours. Median frequency of imaging was 14.5 hours [5.7, 23.9] (**Supplementary Table 1**).

### Longitudinal Trajectory Analyses

**Figure 2:**
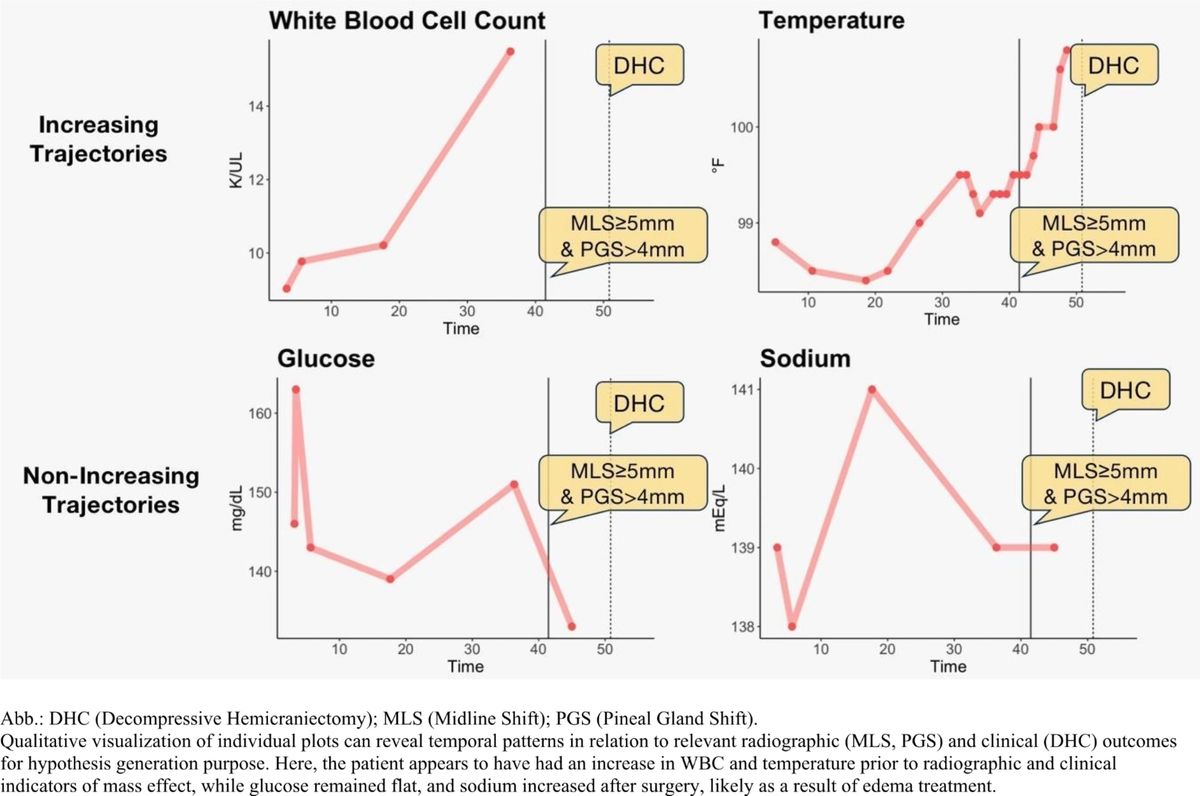
Individual trajectories of longitudinal biomarkers in a single patient leading up to radiographic and clinical evidence of mass effect

We first created univariable trajectories over time for each patient. We developed an online web application in which users can upload baseline and longitudinal data to visualize trajectories related to radiographic and clinical outcomes (https://onglab.shinyapps.io/labvitalplots/) Details about the development of the individual longitudinal plot function are included in the supplementary material. The individual trajectories are “forward looking,” showing the trajectories from the first available measurement until the occurrence of the outcome, death, 192 hours, or end of follow-up, whichever came first. In the results section, we report selected for plots for one patient to illustrate patterns we judged most informative (Fig. 2).

**Figure 3:**
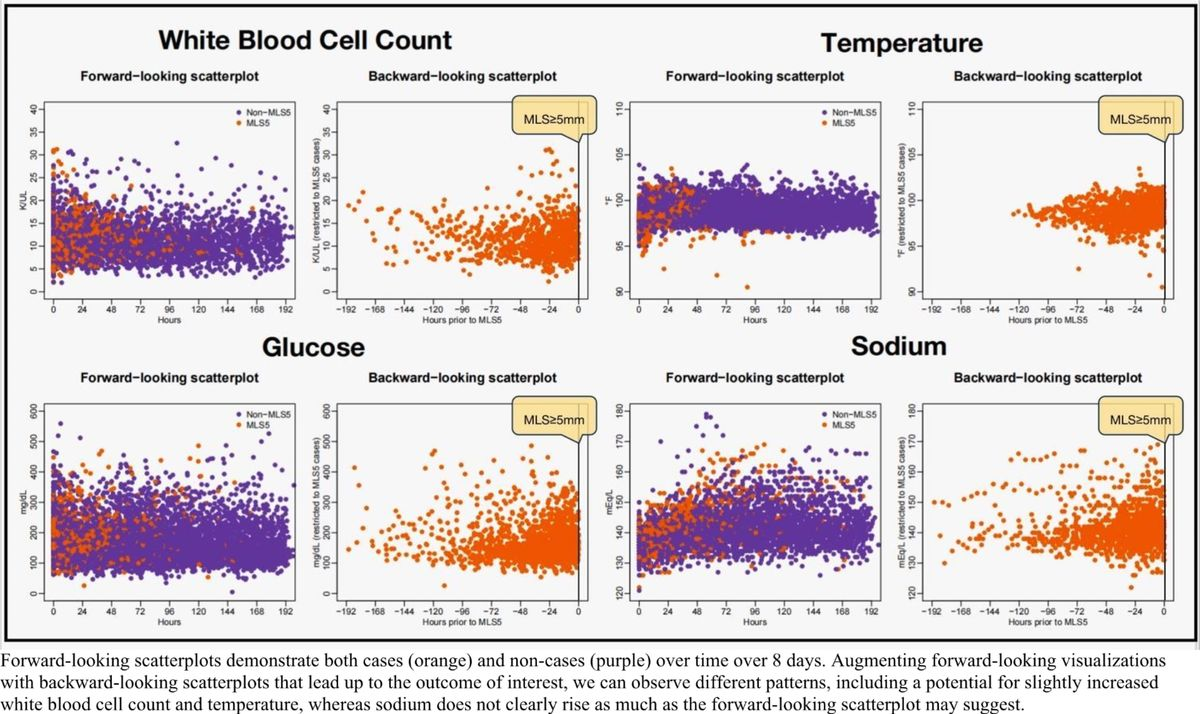
Cohort level scatter plots of longitudinal biomarkers using all data points from ICU hospitalization (forward-looking scatter plot) and a subset of data leading up to midline shift >5mm (backward-looking scatter plot)

We then created scatter plots showing the repeated measurements of longitudinal variables since admission for *all* patients (forward-looking scatter plot) to obtain a global visualization for the 635 patients in our cohort. In patients with our outcomes of MLS ≥5mm, PGS >4mm, and DHC, we also created a backward-looking scatterplot showing the longitudinal variables during the hours leading up to our outcomes (Fig. 3).

We estimated cohort-level univariable trajectories across all patients by using the “backward looking” method developed by Huang and colleagues.^18^ Patients were aligned by using the time of outcome occurrence as the origin and then “look backward” at the distributions of each longitudinal variable prior to that outcome. The outcomes were not observed for all patients in the study cohort and consequently their time origins cannot be identified. However, the “backward looking” method uses longitudinal data from all patients, regardless of whether the outcomes are observed or right-censored. For each variable and each outcome, we estimated the mean trajectory, as well as the median, 25th and 75th percentile trajectories. We then visually examined the shapes of trajectories to identify “signature trajectories,” defined as those with gradual increases or decreases leading up to the outcome, or possibly upward or downward spikes prior to the outcome.

To address measurements taken at irregularly spaced time points in clinical practice, we first identified available data points for every hour interval since admission or stroke onset for blood pressure and heart rate, every two hours for temperature, six hours for glucose and sodium, 12 hours for creatine and radiographic variables (MLS, PGS), and 24 hours for WBC. We then inferred hourly biomarker values for each variable using piecewise linear interpolation for continuous variables and midpoint interpolation for dichotomous variables.^19^ Heatmaps of available longitudinal variables are included in **Supplementary Fig. 1**. All statistical analysis was conducted in R Studio Version 1.3.959 (*RStudio Team (2020). RStudio: Integrated Development for R. RStudio, PBC)* and SAS.

## Results

### Characteristics of cohort patients

Among 1,206 patients with manually verified large ischemic stroke, 635 met all eligibility criteria (Fig. 1). Mean age was 69 ± 15 years, and 311 (49%) were female. In all, 465 (73.2%) patients had pre-existing hypertension, 315 (49.6%) atrial fibrillation, 198 (31.2%) heart failure, and 73 (11.5%) prior stroke. At admission, the mean NIHSS was 17.3 ± 5.9 and ASPECTS was 4.7 ± 3.0. Large vessel occlusion was noted in 303 patients (47.7%). With regards to medication, 285 (44.9%) patients received intravenous thrombolysis, and 123 (19.4%) received mechanical thrombectomy. Two-hundred and twenty two (34.9%) patients experienced MLS ≥5mm prior to DHC, 153 had PGS >4mm (24.1%), and 68 (10.7%) underwent DHC (Table 1). Two-hundred and thirty-nine patients (37.6%) died by discharge. Cumulative incidence plots are included in **Supplementary Fig. 2**. The cumulative incidence of MLS ≥5mm was < 10%, 22%, and 29.4% by 24, 48, and 72 hours respectively. The cumulative incidence of PGS > 4mm was 7, 14, and 20.5% by 24, 48, and 72 hours respectively. The vast majority of DHC 9.6% of 11.3% of all DHCs occurred within the first 72 hours.

In patients with MLS ≥5mm, mean age was 66 ± 14.4, 46.4% were female, and 71.6% were White. Right hemisphere stroke occurred in 57.2%, and average admission ASPECTS was 4.2 ± 3.1 (Table 1). Admission glucose was 156 ± 61 mg/dL in patients with MLS ≥5mm compared with 146 ± 56 mg/dL in those with none. There were no evident differences between patients with MLS <5mm and those with ≥ 5mm in admission WBC, creatinine, or sodium levels, SBP, heart rate, or temperature. One hundred and forty-four (34.9%) of patients with MLS <5mm died by discharge, as opposed to 95 (42.8%) of those with MLS ≥5mm, (41.8%) of those with PGS >4mm and 16 (23.5%) of those with DHC. Full data for patients with PGS >4mm and DHC are included in **Supplementary Table 2.**

For patients with longitudinal vital signs (N = 166) there were no differences in average baseline systolic blood pressure (**SBP**), heart rate, or temperature between groups. Baseline heart rate appeared lower in patients with PGS > 4mm (78.6 ± 22.5) and DHC (76.6 ± 19.8) versus patients who did not develop MLS ≥ 5mm (82.1 ± 18.4) (Table 2). Mean values of longitudinal variables prior to outcomes are included in **Supplementary Table 3.**

### Individual Forward-Looking Trajectories

Figure 2 illustrates dynamic marker trajectories for an individual patient. On visual inspection, WBC and temperature increase leading up to primary and secondary outcomes, while glucose and sodium do not have an upward trajectory on the individual level. Individual patient level trajectories can be constructed from uploaded data, and an example of randomized data is included in the Shiny Application Tool.

### Cohort-Level Backward-Looking Trajectories

Figure 3 compares cohort-level forward-looking scatterplots showing repeated measurement from admission among all patients to backward-looking scatter plots showing measurements leading up to MLS ≥5mm in the subset of patients in which the outcome was observed. In the forward-looking plots, certain biomarkers, like WBC, are widely varied at admission before tapering to a more constrained range, while the backward-looking plot suggests there may be a slight increase prior to outcome. Similarly, the forward-looking scatterplot of temperature appears very stable, while the backward-looking version shows a slight increase. Sodium appears to increase overall throughout admission, which is expected given that sodium is often iatrogenically increased as a management strategy. However, it does not clearly increase prior to the outcome of MLS ≥5mm, but is noticeably denser, likely reflective of increased sodium monitoring, and subsequent increased data points. Cohort-based scatterplots for all variables and outcomes are included in **Supplementary Fig. 3**.

**Figure 4:**
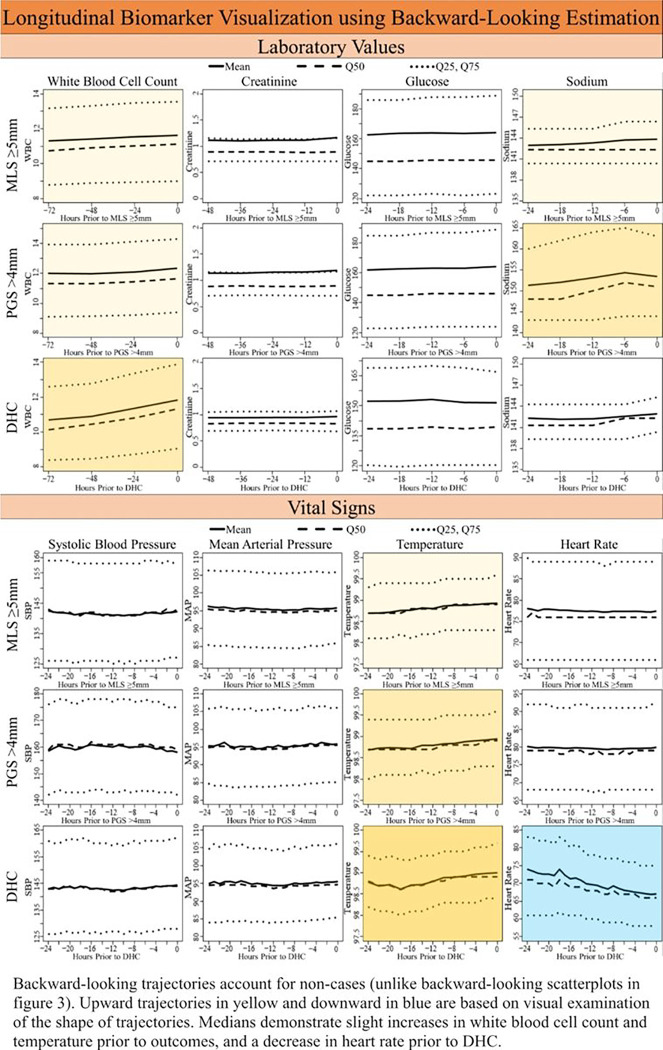
Trajectories longitudinal biomarkers leading up to midline shift >5mm, pineal gland shift >4mm, and decompressive hemicraniectomy using backward look and estimation approach

Figure 4 shows the cohort-level backward-looking trajectories. We observed that WBC and temperature slightly but consistently increased prior to each of the three outcomes, MLS ≥5mm, PGS >4mm, and DHC. Seventy-two hours prior to outcome, median WBC increased a median of 1 K/UL (10–11 K/UL prior to DHC). Within 24 hours prior to outcome temperature increased up to half a degree, from approximately 98.5–99°F. Sodium increased 1–3 mEq/L in the 24 hours prior to MLS ≥5mm and PGS >4mm, and decreased in the six hours prior to DHC. Dynamic trajectories for other variables, including glucose, creatinine, and SBP, were relatively flat prior to all outcomes. Heart rate appeared to decrease from a median of 75 to 68 beats per minute prior to PGS >4mm and DHC (Fig. 4). In our exploratory review of MLS and PGS, we found an expected increasing trajectory from 6–7 mm of MLS 48 hours prior to PGS > 4mm (**Supplementary Fig. 4**).

## Discussion

In this study, we present a multi-faceted approach to examine dynamic physical, laboratory, and radiographic assessments over time in the first several days after large MCA stroke. We curated a unique dataset of 635 patients with granular, repeatedly measured data. We investigated biomarker courses over time in relation to three life-threatening space occupying mass effect outcomes.

In our population we observed small but noticeable increases in WBC and temperature that preceded radiographic and clinical deterioration due to space-occupying mass effect. These findings were revealed by looking at the variables backward from outcome occurrence and were not obvious in conventional forward-looking plots from the time of admission. While the magnitude was small, WBC and temperature trajectories were consistent in directionality and progressively more pronounced for the three outcomes, suggesting that these variables may be markers of increasing severity of space-occupying mass effect. The etiology of the increase in WBC and temperature leading up to radiographic and clinical indicators of space-occupying mass effect findings is unknown. However, potential reasons may be that increased WBC and temperature are reflective of either an increased inflammatory response due to edema itself or that concurrent infection may in fact worsen edema. We suspect that the observation that sodium mildly increases prior to substantial PGS and DHC reinforces the validity of the trajectory modeling approach, as it is often a treatment target in patients with increasing mass effect. We also observed that heart rate declined over the 24 hours prior to DHC. Physiologically, extreme shifts of brain tissue and herniation, which typically occur after MLS ≥5mm, have been associated with classic syndromes including Cushing’s Triad, comprised of bradycardia, hypertension, and irregular respirations.^20^ However, declining heart rates within normal limits (as opposed to frank bradycardia) up to 24 hours prior to clinical deterioration inferred by DHC are not currently recognized as a potential marker for neurologic deterioration.

Our observation that many trajectories were stable may be partially explained by modifying treatments including antihypertensives and insulin given by clinical providers. The absence of an increasing trajectory of glucose, contrary to prior studies that have found that baseline elevated glucose associated with increased edema,^3,17^ may lead investigators to consider whether increasing insulin requirements are a potential clinically relevant biomarker. We did not have access to insulin doses in this cohort. Other possible reasons for stable biomarker values may be because MLS ≥5mm does not sufficiently compress the brainstem to result in changes in serum laboratory values or vital signs.

The backward looking and estimation approach can augment simpler methods that consolidate repeatedly measured data using means or medians over time. Assessing how a biomarker is related to an outcome temporally is important to gain an understanding of phenotypic manifestations of disease progression. The approach can reveal patterns of expression level changes leading up to the event of interest in a way that lessens bias by accounting for repeatedly measured data of censored subjects who did not develop the event and truncating the use of data occurring after the event.

Moreover, our review and constructed tool of longitudinal trajectories over time in single individuals using the R Shiny App enables investigators to examine cases for hypothesis-generating purposes. Our approach of reviewing both forward- and backward-looking data provides a way to preliminarily test hypotheses seen in individual patients.

While variable trends can be incorporated into existing electronic medical record software including EPIC, they often exist as a single longitudinal trend, without adjustment for other factors that may either increase or decrease a patient’s risk of developing complications. Unlike the individualized patient charts we propose, variable trends also lack important intermediate milestones (including radiographic or clinical events) that can better alert clinicians to subtle or signature patterns prior to devastating late-stage events like herniation or neurologic deterioration.

Moreover, it is notable that the median trajectory patterns we observed were subtle, and did not include extremely abnormal values prior to any of our outcomes. We posit that subtle trajectories such as a decrease in beats per minute that remain within normal limits prior to DHC precede late occurring frank abnormalities. If validated, recognition of such trajectories could provide clinicians with the opportunity to obtain confirmatory diagnostic tests and intervene earlier before irreversible secondary catastrophic events.

Finally, as computational and visual tools become increasingly sophisticated, user-friendly, and widely available, we are approaching a time in which *not* systematically using real- or near-real time data to make management decisions will become unacceptable. Foundational work on clinically relevant trajectories will enable the development and validation of dynamic models that update over time with new information, providing more precision based quality care to individuals rather than population averages.

Our study has limitations. Our use of retrospective, observational data may be subject to residual confounding. Moreover, the backward looking modeling requires equally spaced data points. To fulfill this assumption, we assumed latent and linear progression between time points. Progression could theoretically be non-linear or have a different functional form on each time interval. Our cohort comes from two hospitals from a single region in which most of the population is White, and generalizability is uncertain. However, to amass a sufficiently large sample size of patients to better examine risk factors and outcomes, the large inclusion time was necessary. Moreover, the backward-looking estimation is a visualization tool and the shape of the trajectory is prone to subjectivity. However, rigorous methods of reviewing such trajectories must begin at the qualitative stage and are important for hypothesis generation for future, more definitive quantitative study. Finally, our observation of the outcome is dependent on diagnostic imaging, leading to potential nonrandom misclassification (e.g., elevations in WBC may occur after increasing MLS but appears prior due to when imaging was observed). However, given that we have consistent and increasing findings for three different outcomes, we are reassured of the potential veracity of the signal. Future directions include comparison of models that use dynamic predictors to current standards using static information only. Our patients may have been censored by death that occurred prior to outcome due to lack of imaging or withdrawal of life sustaining measures. Patients could be censored by DHC that occurred prior to radiographic outcomes.

Despite the limitations, we report on the largest dataset of MCA stroke patients with rigorously curated longitudinal laboratory, vital sign, and radiographic information of which we are aware. As electronically available repeatedly measured data are increasingly used, it becomes more important to develop robust methods to identify clinically relevant patterns that precede neurologic or systemic deterioration. Our methodologies serve as foundational hypothesis-generating work that can be applied to a variety of life-threatening conditions in the intensive care unit and our univariable findings will be used to inform the development of multivariable dynamic risk prediction models for life-threatening space-occupying mass effect.

## Conclusion

We developed a curated, multi-dimensional, longitudinal dataset for a large cohort of MCA stroke patients. Univariable backward-looking trajectories showed that increasing WBC and temperature may precede radiographic and clinical deterioration due to space-occupying mass effect.

## Figures and Tables

**Table 1:** Baseline Cohort Characteristics

Variable	All N = 635	MLS < 5mm N = 413 (65.0%)	MLS ≥5 N = 222 (35.0%)
Demographics			
Age	68.6 ± 15.3	70.0 ± 15.6	66.0 ± 14.4
Female	311 (49.0%)	208 (50.4%)	103 (46.4%)
Race			
White	490 (77.2%)	331 (80.1%)	159 (71.6%)
Black	41 (6.4%)	21 (5.1%)	20 (9.0%)
Asian	26 (4.1%)	14 (3.4%)	12 (5.4%)
Other^a^	78 (12.3%)	47 (11.4%)	31 (14.0%)
**Past Medical History**			
Hypertension	465 (73.2%)	311 (75.3%)	154 (69.4%)
Atrial Fibrillation	315 (49.6%)	225 (54.5%)	90 (40.5%)
Heart Failure	198 (31.2%)	131 (31.7%)	67 (30.2%)
Prior Stroke	73 (11.5%)	56 (13.6%)	17 (7.7%)
**Home Medication**			
Antiplatelets	205 (32.3%)	143 (34.6%)	62 (27.9%)
Anticoagulants	85 (13.4%)	55 (13.3%)	30 (13.5%)
Statins	196 (30.9%)	126 (30.5%)	70 (31.5%)
**Presentation Information**			
Inpatient Care Prior to Stroke	49 (7.7%)	35 (8.5%)	14 (6.3%)
Right Sided Stroke	329 (51.8%)	202 (48.9%)	127 (57.2%)
Admission ASPECTS			
10 – 8	121 (19.0%)	83 (20.1%)	38 (17.1%)
7 – 4	269 (42.4%)	192 (46.5%)	77 (34.7%)
3 – 0	245 (38.6%)	138 (33.4%)	107 (48.2%)
NIHSS	17.3 ± 5.9	17.0 ± 6.1	18.0 ± 5.4
**Large Vessel Occlusion Location**			
Internal Carotid Artery	134 (44.2%)	79 (42.7%)	55 (46.6%)
M1	117 (38.6%)	72 (38.9%)	45 (38.1%)
M2 or more distal	49 (16.2%)	32 (17.3%)	17 (14.4%)
**Acute Intervention**			
Tissue Plasminogen Activator	285 (44.9%)	184 (44.6%)	101 (45.5%)
**Radiographic Values**			
Hemorrhagic Transformation			
None	268 (42.2%)	227 (55.0%)	41 (18.5%)
Petechial only	309 (48.7%)	171 (41.4%)	138 (62.1%)
Parenchymal	58 (9.1%)	15 (3.6%)	43 (19.4%)
**DHC**	68 (10.7%)	22 (5.3%)	46 (20.7%)
**Death at discharge**	239 (37.6%)	144 (34.9%)	95 (42.8%)

Abb.: ASPECTS (Alberta Stroke Program Early CT Score); DHC (Decompressive Hemicraniectomy); MLS (Midline Shift); NIHSS (National Institutes of Health Stroke Scale); PGS (pineal Gland Shift)

a Other Races, includes American Indian, Alaska Native, Native Hawaiian, Pacific Islander, Not Recorded, Not Given or Unknown.

Missing values: NIHSS = 47 (7.4%).

**Table 2:** Baseline Longitudinal Variables on admission

Variable	All N = 635	MLS < 5mm N = 413 (65.0%)	MLS5 ≥ 5 N = 222 (35.0%)	PGS >4mm N = 153 (24.1%)	DHC N = 68 (10.7%)
**Labs** Glucose (mg/dL)	149 ± 58	146 ± 56	156 ± 61	155 ± 63	154 ± 67
White Blood Cell Count (K/UL)	11.2 ± 4.1	11.2 ± 3.9	11.3 ± 4.4	11.2 ± 4.5	11.2 ± 3.6
Creatinine (mg/dL)	1.1 ± 0.8	1.1 ± 0.8	1.1 ± 0.8	1.2 ± 0.9	1.0 ± 0.3
Sodium (mEq/L)	139 ± 3	138 ± 4	138 ± 3	138 ± 4	138 ± 4
**Vital Signs**	N = 166	N = 101	N = 65	N = 47	N = 30
Systolic Blood Pressure (mmHg)	153 ± 29	152 ± 28	155 ± 31	158 ± 32	159 ± 28
Mean Arterial Pressure (mmHg)	106 ± 19	105 ± 18	107 ± 21	109 ± 21	112 ± 19
Heart rate (beats per minute)	81 ± 20	82 ± 18	80 ± 21	79 ± 23	77 ± 20
Temperature (Fahrenheit)	97.7 ± 1.0	97.7 ± 1.0	97.6 ± 1.0	97.5 ± 1.0	97.6 ± 0.9

Abb.: DHC (Decompressive Hemicraniectomy); MLS (Midline Shift); PGS (Pineal Gland Shift)

Missing values: glucose = 2 (0.3%); WBC = 3 (0.5%); creatinine = 3 (0.5%); sodium = 2 (0.3%); SBP = 1 (0.6%); MAP = 1 (0.6%).
